# Editorial: DNA Replication Origins in Microbial Genomes

**DOI:** 10.3389/fmicb.2015.01545

**Published:** 2016-01-08

**Authors:** Feng Gao

**Affiliations:** ^1^Department of Physics, Tianjin UniversityTianjin, China; ^2^Key Laboratory of Systems Bioengineering, Ministry of Education, Tianjin UniversityTianjin, China; ^3^SynBio Research Platform, Collaborative Innovation Center of Chemical Science and EngineeringTianjin, China

**Keywords:** archaea, bacteria, yeast, replication origin, DNA replication, replication regulation, orisome, regulatory proteins

In all three domains of life, DNA replication initiates on defined genome sites, termed replication origins. In bacteria, replication typically initiates from a single replication origin (*oriC*). In eukaryotic genomes, replication initiates from significantly more replication origins, ranging from hundreds in yeast to tens of thousands in human (Gao et al., 2012). Within the archaeal domain, multiple replication origins have been identified in *Sulfolobus* species, haloarchaea etc. (Lundgren et al., 2004; Robinson et al., 2004; Wu et al.; Yang et al., 2015). The research on replication origins is important not only in providing insights into the structure and function of the replication origins but also in understanding the regulatory mechanisms of the initiation step in DNA replication. Therefore, intensive studies, by *in silico* analyses as well as *in vivo* and *in vitro* experiments, have been carried out in the last two decades.

Based on the sequence-derived features, various *in silico* approaches have been developed to identify microbial replication origins (Frank and Lobry, 2000; Breier et al., 2004; Mackiewicz et al., 2004; Zhang and Zhang, 2005; Worning et al., 2006; Gao and Zhang, 2007, 2008a; Gao et al., 2013; Gao, 2014). For example, the locations of replication origins sites have been predicted for thousands of bacterial genomes by Ori-Finder, a web-based system for finding *oriC*s in bacterial genomes (Gao and Zhang, 2007; Gao et al., 2013). A new version of Ori-Finder for archaea, Ori-Finder 2, has been developed to predict *oriC*s in archaeal genomes automatically (Luo et al.). To confirm the predicted replication origins, it is important to choose a most suitable experimental strategy. Song et al. summarize the main existing experimental methods to determine the replication origin regions and their practical applications (Song et al.). As a study from *in silico* to *in vitro*, the experimental supports are provided for the identified replication origins in *Cyanothece* ATCC 51142 (Gao and Zhang, 2008b), and their interactions with the initiator protein DnaA (Huang et al.).

In spite of a great variety of origin sequences across species, all bacterial replication origins contain the information necessary to guide assembly of the DnaA protein complex at *oriC*, triggering the unwinding of DNA and the beginning of replication. Therefore, *oriC*-encoded instructions should be interpreted particularly in the context of replication initiation and its regulation (Wolanski et al.). Wolanski et al. show that *oriC*-encoded instructions allow not only for initiation but also for precise regulation of replication initiation and coordination of chromosomal replication with the cell cycle (also in response to environmental signals; Wolanski et al.). Frimodt-Moller et al. find control regions for chromosome replication are conserved with respect to sequence and location among *Escherichia coli* strains (Frimodt-Moller et al.). Based on the single origin usage strategy that distinguishes bacteria, Marczynski et al. redefine the bacterial origins as centralized information processors, and describe how negative-feedback, phospho-relay, and chromosome-partitioning systems act to regulate chromosome replication (Marczynski et al.). On the other hand, the *in silico* analyses show that some bacteria, although very few, may have multiple origins of replication per chromosome (Frank et al., 2015; Gao), and the recent work also suggests that there are multiple replication origins in *Synechocystis* that fire asynchronously, as in eukaryotic nuclear chromosomal replication (Ohbayashi et al., 2015).

For eukaryotic organisms, replication origins are best characterized in the unicellular eukaryote budding yeast *Saccharomyces cerevisiae* and the fission yeast *Schizosaccharomyces pombe*. With the recent development of genome-wide approaches, the number of yeast species involved in ORIs research has increased dramatically, which has created opportunities for the sequence, protein, and comparative analysis of replication origins in yeast genomes (Li et al.; Zheng et al.; Peng et al.).

The Frontiers in Microbiology Research Topic on DNA replication origins in microbial genomes is devoted to address the issues mentioned above, and aims to provide a comprehensive overview of the current research in this field.

## Dedication

This article is dedicated to the 120th Anniversary of Tianjin University (formerly Peiyang University), the first modern higher education university in China.

### Conflict of interest statement

The author declares that the research was conducted in the absence of any commercial or financial relationships that could be construed as a potential conflict of interest.
